# Preliminary and Ongoing Investigation of Human Induced Pluripotent Stem Cell‐Derived Frontotemporal Dementia Cortical Organoids Assessed by Mass Spectrometry

**DOI:** 10.1002/alz70855_102339

**Published:** 2025-12-23

**Authors:** Merci N Best, Jared Lamp, Andrew Umstead, Mariana Sierra, Amber Spencer, Ilkim Erturk, Elizabeth M Tank, Jonathan Sexton, Sami Barmada, Irving E Vega, Henry L Paulson

**Affiliations:** ^1^ University of Michigan, Ann Arbor, MI, USA; ^2^ Michigan State University, Grand Rapids, MI, USA; ^3^ Michigan Alzheimer's Disease Research Center, Ann Arbor, MI, USA

## Abstract

**Background:**

Tau dysfunction occurs in over 20 diseases, including frontotemporal dementia. The well‐characterized frontotemporal dementia‐associated *MAPT* ^V337M^ variant is a missense single nucleotide mutation that results in the substitution of valine with methionine at position 337 in the gene that encodes tau protein. Tau phosphorylation is increased in human induced pluripotent stem cell‐derived *MAPT* ^V337M^ cortical neurons and organoids. However, the impact of *MAPT* ^V337M^ on the proteomic profile of organoids has not been previously explored.

**Method:**

We generated single‐rosette organoids from three isogenic human induced pluripotent stem cell lines: *MAPT* ^WT/WT^ (wild type), *MAPT* ^WT/V337M^ (heterozygous), and *MAPT* ^V337M/V337M^ (homozygous) provided through the generous support of the Tau Consortium of the Rainwater Charitable Foundation. These organoids were cultured for 90 and 180 days. To assess protein levels alongside posttranslational modifications, we performed protein identification and label‐free quantitation on 18 organoids (three organoids from each genotype and time point). Database searching and protein identification were performed by Proteome Discoverer 2.5.0 via SEQUEST against the reviewed *Homo sapiens* database (UP000005460) including trypsin, Lys‐C, contaminant sequences, and *MAPT* ^V337M^.

**Result:**

Label‐free quantitative analysis identified 2,988 proteins, revealing significant differences across genotypes and time points. Time had a more profound effect on protein composition than genotype. Tau was observed in all samples and over 25 phosphorylation sites on tau were detected. 180‐day organoid samples displayed increased peptides in the 4R tau domain. Heterozygous organoids deviate more strongly from wild type than homozygous organoids, but significant protein changes can be found in both sample groups. Of these significant protein changes, we are working to validate increases in heterozygous and homozygous organoids annexin A11 (ANXA11) at 90 days and clusterin (CLU) and tubulin polymerization promoting protein family member 3 (TPPP3) at 180 days.

**Conclusion:**

This study leverages human induced pluripotent stem cell‐derived *MAPT* ^V337M^ cortical organoids as a model to explore frontotemporal dementia‐associated proteomic changes. The observation that time impacts protein composition more than genotype suggests the importance of temporal dynamics in organoid development. This study serves as a template for examining other genetic mutations linked to dementia.

